# Novel Therapeutics for Multiple Sclerosis Designed by Parasitic Worms

**DOI:** 10.3390/ijms18102141

**Published:** 2017-10-13

**Authors:** Aakanksha Dixit, Akane Tanaka, Judith M. Greer, Sheila Donnelly

**Affiliations:** 1 The University of Queensland, UQ Centre for Clinical Research, Brisbane, QLD 4029, Australia; a.dixit@uq.edu.au (A.D.); j.greer@uq.edu.au (J.M.G.); 2 The School of Life Sciences, University of Technology Sydney, Ultimo, NSW 2007, Australia; akane.tanaka@uts.edu.au

**Keywords:** multiple sclerosis, environmental factors, helminth parasites, old friend’s hypothesis, immune modulation, innate immunity

## Abstract

The evolutionary response to endemic infections with parasitic worms (helminth) was the development of a distinct regulatory immune profile arising from the need to encapsulate the helminths while simultaneously repairing tissue damage. According to the old friend’s hypothesis, the diminished exposure to these parasites in the developed world has resulted in a dysregulated immune response that contributes to the increased incidence of immune mediated diseases such as Multiple Sclerosis (MS). Indeed, the global distribution of MS shows an inverse correlation to the prevalence of helminth infection. On this basis, the possibility of treating MS with helminth infection has been explored in animal models and phase 1 and 2 human clinical trials. However, the possibility also exists that the individual immune modulatory molecules secreted by helminth parasites may offer a more defined therapeutic strategy.

## 1. Introduction

Multiple Sclerosis (MS) is an autoimmune demyelinating disease that can manifest as a wide range of clinical signs and symptoms and present with varying severity. It is generally accepted that a combination of individual genetic susceptibility and environmental factors contribute to the development of autoimmune diseases like MS, although the relative contribution of each of these factors is hotly debated [1,2].

Genome wide association studies (GWAS) of MS patients have identified over 100 genetic risk loci. However, most of these exert only a modest influence on MS risk and contribute only 20–30% of the perceived heritability of MS, suggesting that the remaining 70–80% of unexplained susceptibility is affected by environmental factors [2,3,4]. Furthermore, in studies of identical twins where one develops MS, only 25% of second twins developed the disease [5]. Such discordance between monozygotic twins strongly supports a critical role for an environmental factor in the initiation of MS. However, perhaps the most compelling evidence in this regard has been the rapid increase in the incidence rates of MS in the developed world over recent years [6,7,8,9]. This rise far exceeds the rate of population evolution and therefore implies either the removal of protective factors or the introduction of susceptibility factors in the environment. Indeed, population migration studies indicate that the place of residence in adolescence or early adulthood strongly influences a person’s subsequent risk of MS [10,11,12,13].

Identification of the possible environmental factors has been informed by the unusual geographic distribution of the disease. The prevalence of MS increases with increasing latitude, such that higher rates of incidence are found in high latitude regions [4,14]. MS is also more common in the developed world than the developing world [15]. Based on these epidemiological observations, several putative environmental risk factors have been proposed such as low vitamin D, Epstein–Barr virus, sanitation and smoking [4,15,16,17]. However, perhaps the most compelling environmental relationship is the worldwide inverse correlation between infections with parasitic worms (helminths) and the incidence of autoimmune disease [18].

Corroborating this hypothesis, longitudinal and migratory studies evaluating the prevalence of MS in the French West Indies over a period of 20 years showed that increased MS incidence in the region was associated with a significant reduction of parasite infections during the same time period [19]. More specifically, the prevalence of MS was reportedly significantly reduced once a critical threshold (10%) of infection with the helminth *Trichuris trichiura* was exceeded in any given population [20]. The fact that administration of anti-helminth drugs resulted in increased MS activity [21], suggests that helminths directly suppress autoimmune diseases and may be the protective environmental factor against the development of MS.

## 2. Should We Reunite with Our Old Friends the Helminth Parasites?

The human immune system evolved to provide protection from a range of pathogenic microorganisms (viruses, bacteria, fungi and protozoan). However, despite also being regarded as pathogenic, the immune response to helminth parasites does not typically provide protection from re-infection. Instead, most of these organisms are well tolerated by their human hosts, with infections lasting for many years [22]. Moreover, infections are primarily asymptomatic and mortality is rare. It is suggested that this phenomenon has occurred due to the long evolutionary coadaptation between these parasites and man [23,24], through which helminth parasites have strongly influenced the evolution of the human immune system. This is because infection was not associated with the onset of epidemics but rather helminth parasites have been ubiquitous within human populations over millennia [23].

The canonical immune response to helminth parasite infection is the development of an anti-inflammatory, type 2 immune response, characterised by the secretion of cytokines Interleukin (IL)-4, IL-5, IL-9 and IL-13 [25,26,27], and the simultaneous suppression of pro-inflammatory T helper (Th)-1 and Th17 responses [28]. Initially, it was thought that these type 2 cytokines were produced by Th2 cells. However, several studies have demonstrated that the helminth-induced type 2 responses can be generated in the absence of T cells [29] and have led to the recent identification of a non-redundant role for type 2 innate lymphoid cells (ILC2) in the recognition and response to parasitic worms [30]. ILC2 secrete IL-4, IL-5, IL-13 and amphiregulin to combat helminth infection [31]. This switch towards a type 2 immune response also leads to the inhibition of pro-inflammatory M1 macrophages and dendritic cells (DCs) and, instead, the induction of an immature phenotype of dendritic cells (DC2s) and an anti-inflammatory phenotype of macrophage (termed helminth-M2 macrophages) [32]. As the parasite infection reaches chronicity, the differentiation of T-regulatory (Treg) populations is initiated. These cells secrete regulatory cytokines, such as IL-10 and transforming growth factor-β (TGF-β) [33], which further inhibit Th1 responses, and also regulate type 2 responses. This prevents the generation of extensive tissue fibrosis, which would result from sustained wound healing activity mediated by helminth-M2 macrophages and their products [34].

The outcome for the host is to create a balance in which the parasite is tolerated, and homeostasis can be maintained. It is suggested that the Treg response is crucial for invoking specific immunological tolerance. However, when damage does occur, a more physiological tolerance mechanism, including the ability to repair the damage, is maintained by type 2 effector pathways [35].

This evolutionary development in immune responsiveness to infection with helminths has resulted in compensatory adjustments to immune-related genes in human populations [36,37]. In fact, it has been proposed that, in order for the human immune system to operate optimally, the presence of helminth induced immunoregulatory networks are required [38]. Accordingly, in populations where parasitic infections are no longer endemic, there is an increased likelihood of inappropriate immune responsiveness to autoantigens, and the concomitant development of autoimmune/inflammatory diseases, such as MS. Consequently, many have asked the question of whether helminths should be regarded as beneficial commensals and whether the re-introduction of these old friends may be therapeutic in MS [7,39,40,41,42,43].

## 3. Worm Therapy: Proof-of-Principle in Experimental and Clinical Trials

### 3.1. Testing the Effectiveness of Helminth Infection in Animal Models of MS

The most widely used animal model of MS is experimental autoimmune encephalomyelitis (EAE), which mimics several of the key clinical and pathological features of the human disease, such as paralysis and demyelination. EAE studies have provided critical information on autoimmune related central nervous system (CNS) damage, demyelination, presence of immunoglobulins in CNS and cerebrospinal fluid, distribution of lesions and remyelination processes, and much of our understanding of the likely pathological mechanisms in MS came from work using the EAE models.

EAE is actively induced in rodents via immunisation with a neuroantigen emulsified in adjuvant (typically complete Freund’s adjuvant). The neuroantigen is typically a whole tissue homogenate (e.g., spinal cord), myelin protein (e.g., MOG, myelin oligodendrocyte glycoprotein), or purified myelin peptide (e.g., MOG_35–55_, PLP_139–151_). The experimental choice of animal, neuroantigen and adjuvant influences the outcome of EAE, which is quite variable (e.g., acute, relapsing, or chronic forms) [44]. After immunisation with the neuroantigen, there is typically an induction phase (0–10 days after immunisation) during which the autoreactive T cells are activated and disseminate. This is followed by the effector phase (10–30+ days after immunisation with neuroantigen) during which the autoreactive T cells in the CNS activate microglia and macrophages, which subsequently damage myelin and other CNS components. At this stage, signs of clinical disease, such as paresis and paralysis, are evident in the animals.

Encouragingly, of the 12 studies reported to date (Table 1), in all but one case, infection of rodents with helminth parasites protected the animals from EAE. The range of experimental design, dosing, treatment schedules and choice of helminth (and thus tissue site being colonised) make it challenging to determine a single specific mechanism of disease prevention. However, as expected for a helminth infection, in all cases, there was an overall switch in the autoantigen specific immune response from a pro-inflammatory Th1 and Th17 response towards a type 2 or Treg predominant response. In the majority of cases, the worms were administered to animals prior to the induction phase of disease (pre-induction), which, considering the parasite’s ability to suppress pro-inflammatory immune responses, may simply reflect a suppression of the induction of immune responses to the administered neuroantigen. Alternatively, it could also be considered that such timing for therapeutic manipulation during EAE experiments best models the effect of preventive therapy for a new relapse or episode in MS [15]. More persuasive is the demonstration that, when delivered during the effector phase of the disease, the intestinal parasite *H. polygyrus*, mediated complete amelioration of the clinical signs of disease within six days [45]. In contrast, infection with the blood fluke *S. mansoni* was ineffective when delivered during the effector phase, despite showing efficacy when administered during the pre-induction or induction phases [46]. This suggests that not every helminth will offer beneficial effects and the choice of parasite may be the most critical factor in the development of worm therapeutics.

### 3.2. Testing the Effectiveness of Helminth Infection in Human Trials

The first direct evidence that helminth infection could prevent MS came from a series of observational studies beginning in 2007 [47]. This prospective investigation examined the clinical status of 12 relapsing-remitting MS subjects who had naturally contracted gastrointestinal parasites and compared them to a second cohort of demographically-matched MS patients without parasite infection. Over a period of 4.5 years, the patients infected with helminth parasites had a dramatic and sustained reduction in clinical, magnetic resonance imaging (MRI), and immunological parameters of MS activity, compared to the uninfected subjects. During the follow up study over the next 5–7 years, one third of the patients infected with helminths developed severe gastrointestinal symptoms and were treated to clear the helminth [21]. Unfortunately, elimination of the parasites in those patients also resulted in a return of the clinical and MRI signs of disease to a level similar to that seen in the patients who were not infected. This finding provided support for the notion that helminth infection might be a useful therapeutic approach for the treatment of MS, and led to the initiation of several clinical trials using live helminth infection in patients with MS (summarized in Table 2).

To date, all phase 1 studies have shown that treatment with the helminths is safe. Some, but not all, have also shown promising effects on clinical outcomes, brain MRI measures. However, as efficacy was not the primary outcome of these studies, these data should be interpreted with caution. Over the last three years, two double-blinded, placebo-controlled phase 2 trials have been undertaken. One of these is still ongoing (HINT2) and although the second (WIRMS) is completed; the results have not yet been published. These studies will provide a comprehensive and meaningful outcome and determine the future of live worm therapeutics.

Analysis of systemic immunological changes in MS patients therapeutically infected with helminths reported a profile of beneficial immune modulation. A general increase in serum IL-4, IL-5 and IL-10 supported a helminth induced switch to Th2/Treg immune responses [48]. In addition, the secretion of IFNγ from antigen stimulated T cells was significantly reduced, indicating a suppression of autoantigen specific Th1 responses [49]. A similar outcome has been reported in patients with inflammatory bowel disease and coeliac disease therapeutically infected with *T. trichiura* or hookworm, respectively [50,51]. In both cases, during parasite infection, the clinical symptoms of immune mediated disease abated in parallel with a decrease in IL-17 producing T cells. However, in these particular disease indications, the immune modulation promoted by parasite infection also mediated physiological changes to improve clinical symptoms. The accumulation of IL-22 producing T cells in the intestinal mucosa of infected patients was suggested to promote intestinal mucus production to reduce symptomatic colitis [50]. While similar relationships have not yet been investigated for helminth infected MS patients, given the growing evidence for functional crosstalk between the gut and the CNS, it is possible that intestinal immunological changes occurring in response to helminth infection is causally related to a positive modulation of brain autoimmunity.

## 4. Translating to the Clinic: Worms or Molecules?

Collectively, the evidence gathered from natural endemic population studies, experimental animal work and human trials clearly support the possibility that helminths could be used as a treatment option for multiple sclerosis and provides a strong rationale that “worm therapy” is worth pursuing for the prevention or treatment of autoimmune disease.

However, the use of live parasites is not optimal. The compliance of patients is predicted to be generally poor, and this is largely attributable to negative attitudes associated with the concept of being infected with live pathogens [61]. In addition, the intake of live parasites (and, in some cases, the subsequent infection) has caused adverse side effects, at times even exacerbating pro-inflammatory conditions, due to the parasite’s feeding and migratory activities [62,63].

The larval stages, which have been shown to be therapeutically beneficial in trials, must be produced in a mammalian host [28], so the possibility of a large scale production in compliance with good manufacturing practices (GMP) is limited and prohibits the mass scale production required for a truly global therapeutic. There is also a significant risk of contamination with other human pathogens. For example, the parasite *N. americanus* is harvested from the faeces of infected individuals. The use of the pig whipworm, *T. suis*, does not circumvent this problem, as there is a transferral risk of hepatitis E virus, which is often found in pigs and is pathogenic to humans [64]. Accordingly, the use of pathogen-free pigs would be required to ensure the absence of such infectious contaminants [64,65]. Additionally, using *T. suis* as a therapeutic agent requires the frequent intake of 2500 eggs, a dosing regimen with which many patients fail to comply. This is compounded with the fact that all ova might not hatch into active juveniles, thereby reducing the impact of the dose. Thus, the selection of the right helminth with the exact dosage and schedule of the treatment is difficult.

Finally, but most importantly, there is a lack of specificity with the use of live parasites. In populations endemically infected with helminths, Th1 and/or Th17 immune responses are compromised, which prevents the development of appropriate immune responses to bacterial/viral pathogens and vaccination [66]. Thus, the patient is effectively immune-compromised, just as he/she would be if prescribed immunosuppressive drugs. For individuals who are already immune compromised (such as children, the elderly, and pregnant women), treatment regimens using live helminth infections may be of greater risk than benefit [67,68,69].

A safer and more reliable alternative to live infection would be to identify the specific immune-modulatory molecules produced by helminth parasites. This approach would allow the precise mechanism(s) of action to be characterized, and their properties to be modified to increase therapeutic efficacy. In addition, synthetic proteins can be easily manufactured to therapeutic standard, and can be modified during synthesis into small molecule analogues to enhance stability, and to reduce undesirable immunogenicity and toxicity.

Within their mammalian hosts, helminth parasites secrete and excrete a mixture of molecules. These excretory/secretory (ES) products perform a number of biological functions to support the parasite’s growth and reproduction, such as degradation of haemoglobin into essential nutrients and cleavage of collagen to facilitate migration through tissues. However, the ES products from many helminths also have the capacity to modulate host immune responses, analogous to live infection [70,71,72,73]. Furthermore, the delivery of ES products from a number of different helminths prevents or ameliorates the clinical signs of autoimmune disease in animal models of EAE (Table 3). In these experiments, all of the ES products prevented the development of disease when delivered during the pre-induction phase. Moreover, a strong protective effect was also observed when ES (in two experiments) was administered at or after the induction phase of disease. Consistent with the observation that intraperitoneal delivery of *S. mansoni* eggs to animals afforded protection from EAE (Table 1), the administration of secreted egg antigens (SEA) derived from the same parasite also suppressed the progression of disease (Table 3) [74].

Based on this evidence, a typical process for drug discovery would involve a rational search through the contents of SEA to identify the key molecule that is mediating beneficial immune modulation to prevent disease development. However, SEA is the soluble products derived from the homogenate of eggs and therefore contains numerous components that are naturally secreted from eggs during infections, but also molecules that are normally held within the structure of the egg. Proteomic analysis using two-dimensional electrophoresis of SEA has identified >1000 proteins [75]; thus, identifying the singular components that may be mediating protection from EAE presents a challenging task.

The clinical signs of EAE were also reduced in mice treated with the ES derived from *Fasciola hepatica*. Unlike SEA, this preparation contains only the products that are naturally secreted by the adult worm and therefore is more likely to contain the proteins that are secreted during infection and which interact with the cells of the host immune response. Furthermore, in contrast to the complexity of SEA, proteomic analysis of *F. hepatica* ES has identified 160 proteins, 67% of which are cysteine proteases [76].

## 5. The Peptide FhHDM-1, Secreted by *Fasciola hepatica*, Shows Therapeutic Efficacy in EAE

*F. hepatica*, commonly known as the liver fluke, has adapted to long-term survival within its mammalian hosts, living up to 20 years in some instances [82,83]. Additionally, *F. hepatica* exhibits a widespread geographical distribution, and infects the greatest variety of mammals of all helminth parasites [84,85]. Not only does this imply a superior adaptation, as compared to other helminth parasites, such as *N. Americanus* and *T. suis*, which have adapted to infect only a single host, but it also suggests that Fasciola parasites have evolved universal processes of invasion, virulence and immune modulation.

**Figure 1:**
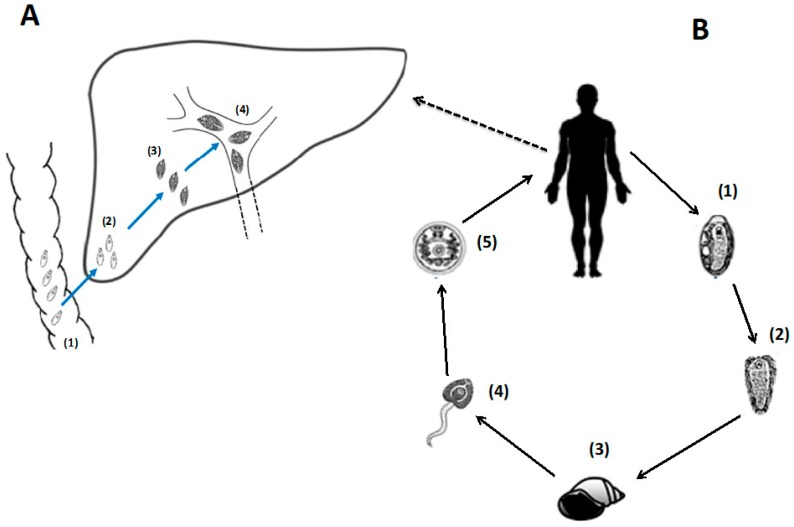
*Fasciola hepatica* lifecycle. Graphical representation of the *F. hepatica* lifecycle (adapted from [82]). (**A1**) parasite excyst in the intestine of the mammalian host, releasing newly excysted juveniles (NEJ) that migrate across the intestinal wall, through the peritoneal cavity to the liver (**A2**). NEJ migrate through the liver parenchyma, increasing in size to juvenile flukes as they migrate into the bile ducts (**A3**), where they grow and develop into fully mature adults (**A4**); (**B1**) eggs are released in the faeces and develop on vegetation; (**B2**) a single miracidium hatches from each embryonated egg and then (**B3**) infects the intermediate snail host (*Galba truncatula*); (**B4**) within the snail, the parasite develops through the sporocyst, rediae and cercariae lifecycle stages. Cercariae are released from the snail and (**B5**) encyst on vegetation as dormant metacercariae, which are subsequently ingested by the definitive host.

The infection pathway of *F. hepatica* begins within the mammalian host after the ingestion of metacercariae, which are the encysted larval form of the worm (Figure 1). Upon reaching the acidic environment of the gastrointestinal tract, immature flukes hatch, and these newly excysted juvenile (NEJ) flukes migrate through the gut wall to enter the peritoneal cavity. The NEJ flukes continue to burrow through to the liver, reaching the bile ducts 10–12 weeks after ingestion. Within the bile ducts, the NEJ flukes mature into adult forms, whose primary activities are feeding on blood by puncturing holes in the bile duct wall, and production of eggs [82]. The mature adults are obligate blood feeders and secrete copious amounts of cysteine proteases (FhCL) to effectively digest blood proteins, particularly haemoglobin, to provide sufficient nutrients required for egg production [86]. Related to this activity is the production of the anti-oxidant peroxiredoxin (FhPrx), which detoxifies the reactive oxygen generated by cellular metabolism [86,87], and the production of FhHDM-1, a peptide that interacts with heme, a toxic by product of haemoglobin digestion [88]. In addition to these activities to support the parasite’s life cycle, these three proteins have also been shown to act as potent immune modulators, specifically inhibiting the activation of pro-inflammatory innate immune responses [89,90,91,92,93].

Considering their capacity to alter inflammatory immune responses, we assessed the therapeutic efficacy of recombinant forms of the three most abundant molecules of *F. hepatica* ES, FhPrx, FhCL1 and FhHDM-1 in an animal model of relapsing remitting EAE. Despite their evident immune modulating activity, neither FhPrx (Donnelly et al., unpublished data) nor FhCL1 [94] prevented disease development. However, a short time course of treatment with FhHDM-1 significantly reduced the clinical signs of EAE [94], which was associated with the absence of immune cell infiltrates into the CNS. These observations suggested that, within the ES of the adult *F. hepatica*, FhHDM-1 may be the sole molecule with potential as an immune therapeutic.

Unexpectedly, FhHDM-1 treatment of mice did not alter the profile of cytokines produced by T cells in response to autoantigen. Unlike infection and the administration of ES, Th1 cytokines were evident and there was no significant switch to a type 2 immune response. However, after parenteral administration, the FhHDM-1 peptide interacted with macrophages and reduced their capacity to secrete pro-inflammatory cytokines. Thus, it has been hypothesized that the inhibition of innate pro-inflammatory immune responses, which are central to the development of autoimmunity in EAE and MS, prevented the trafficking of autoreactive lymphocytes from the periphery to the site of autoimmunity and thus prevented tissue destruction.

## 6. Conclusions

The studies discussed here represent a significant body of evidence that proteins secreted by helminth parasites offer a unique resource for the discovery of anti-inflammatory drugs. The specific finding that a single parasite secreted protein, FhHDM-1, can suppress the pathology associated with EAE, although preliminary, suggests that understanding the mode of action of this parasite protein may provide a novel starting point in the development of safe effective drugs for treatment of human MS. The efficacy of parasite-derived molecules has been fine-tuned over millennia of co-evolution with humans, suggesting that the pharmacological activity of these proteins has already been optimized over time by nature. Therefore, exploiting the excretory secretory products from helminth parasites offers the potential for drug treatments that are far superior to current biologicals whose widespread use is limited by a lack of specificity and a range of adverse side effects.

## Figures and Tables

**Table 1:** Studies reporting live helminth infection in animal models of Experimental Autoimmune Encephalomyelitis.

Animal Model ^a^	Helminth Treatment ^b^	Effects When Treatment Administered at ^c^			Mechanism	Ref.
		Pre-Induction	Induction	Effector		
PLP_139-151_ SJL mice	*Schistosoma mansoni* 5000–20,000 eggs i.p.	++	+	-	Polarisation of immune response to Th2	[46]
	*Schistosoma mansoni* 5000–10,000 eggs i.p./s.c.	++	++	-	Increase in IL-4, reduction in Interferon (IFN)-γ	[46]
MOG_35-55_C57BL/6 mice	*Schistosoma mansoni* 5000–10,000 eggs i.p./ s.c.	++	++	-	Increase in IL-4, reduction in IFN-γ	[46]
	*Schistosoma mansoni* 70 cercariae cutaneous	++	n.d.	n.d.	Reduction of Th1 pro-inflammatory cytokines	[52]
	*Fasciola hepatica* 10 metacercariae p.o.	++	n.d.	n.d.	Bystander attenuation of Th17 and Th1 responses by means of Transforming growth factor (TGF)-β	[33]
	*Trichinella pseudospiralis* 200 larvae p.o.	++	n.d.	n.d.	Strong Th2 responses; Th1 and Th17 responses suppressed	[53]
	*Taenia crassiceps* 400 metacestodes i.p.	+++	n.d.	n.d.	Anti-inflammatory cytokine environment leads to reduced T cell activation	[54]
	*Heligmosomoides polygyrus* 200 larvae p.o.	n.d.	n.d.	++	Reduction of IL-17A, IL-12 and promotion of regulatory cytokines	[55]
MBP-EAE in Lewis rats	*Strongyloides venezuelensis* 4000 larvae s.c.	-	n.d.	n.d.	No effect on disease course; The host may be resistant to helminth infection	[45]
Spinal cord homogenate in Dark agouti (DA) rats	*Trichinella spiralis* larvae Multiple doses by gastric inoculation	500 & 1000 ++	n.d.	n.d.	Th2 response and regulatory response increase, also increase in IL-10	[56]
	*Trichinella spiralia* Larvae p.o.	+	++	n.d.	Th2 cytokine bias; anti-inflammatory responses likely due to regulatory T cells	[57]

^a^ PLP, proteolipid protein; MOG, myelin oligodendrocyte glycoprotein; MBP, myelin basic protein; ^b^ p.o., oral administration; i.p., intra-peritoneal injection; s.c., sub-cutaneous injection; ^c^ The effect of helminth treatment on EAE severity, depending on the time at which helminth was administered is indicated by the following symbols: -, no effect; +, mild; ++, moderate; +++, strong; n.d., not done.

**Table 2:** Human clinical trials of live helminth infection as a therapeutic for Multiple Sclerosis.

ClinicalTrialsgov ^a^	End Date	Subjects ^b^	Helminth Treatment ^c^	Clinical Evaluation Parameters ^d^	Results	Status ^e^	Ref.
HINT 1 NCT00645749	2011	5 RRMS	2500 TSO orally 2 weeks × 12 weeks	Number of new gadolinium enhancing lesions on serial MRI scans (monthly)	Treatment was safe. No adverse effects. No. of lesions fell from 6.6 at baseline to 2. Serum IL-4, IL-10 increased in 4 patients	C	[48]
TRIMS-A 2010 NCT01006941	2011	10 RRMS	2,500 TSO orally 2 weeks × 12 weeks	No. of new or enlarging T2 lesions, no. of Gd enhancing lesions, volume of T2 lesions	Well tolerated; minor gastrointestinal symptoms. No beneficial effect.	C	[58]
Charite safety study	2011	4 SPMS	2500 TSO orally 2 weeks × 4 weeks	Immunological & clinical parameters were assessed	Treatment was safe. Lower Th1 & increase in Th2 (IL-4)	C	[49]
TRIOMS 2012 NCT01413243	2016	50 RRMS	2,500 TSO orally 2 weeks × 12 weeks	Number of new gadolinium enhancing lesions on serial MRI scans; Vol of new T2 hyperintensive in cerebral MRI	Terminated	T	[59]
WIRMS 2011 NCT01470521	2016	72 RRMS	25 live *Necator americanus* dermally	Number of new gadolinium enhancing lesions on serial MRI scans (at month 9), change in expanded disability status scale	Final results not released—expecting lower number of lesions	C	[39]
HINT 2 NCT00645749	2017	18 RRMS	2,500 TSO orally 2 weeks × 10 months	Number of new gadolinium enhancing lesions on serial MRI scans (monthly)	Safety confirmed. Interim MRI and immunological measures positive.	O	[15,60]

^a^ Clinicaltrials.gov website provides details of study design and updates on study progress. (NCT = national clinical trial; HINT = helminth-induced immunomodulation therapy; TRIMS-A = *Trichuris suis* ova therapy for multiple sclerosis—a safety study; TRIOMS = *Trichuris suis* ova in Recurrent Remittent Multiple Sclerosis and Clinically Isolated Syndrome; WIRMS = worms for immune regulation of multiple sclerosis). ^b^ The number and type of MS patients are listed (RRMS = relapsing-remitting MS; SPMS = secondary progressive MS). ^c^ TSO, *Trichuris suis* OVA. ^d^ MRI, magnetic resonance imaging. ^e^ Study status is indicated by C (completed), O (Ongoing), T (Terminated).

**Table 3:** Experimental studies assessing the protective effect of helminth soluble products in animal models of EAE.

Animal Model	Helminth Treatment ^a^	Time Point of Administration ^b^		Mechanism of Protection	Ref.
		Pre-Induction	Induction		
MOG_35-55_ in C57BL/6 mice	*Schistosoma japonicum* SEA 100 μg i.p. once a week for 4 weeks	+	++	Th2 environment established.	[74]
	*Trichuris suis* SP 100 μg i.p. once a week for 4 weeks	++	n.d.	Unknown. In vitro suppression of pro-inflammatory dendritic cells	[77]
	*Trichuris spiralis* SP 100 μg i.p. once a week for 4 weeks	++	n.d.	Unknown. In vitro suppression of pro-inflammatory dendritic cells	[77]
	*Trichuris suis* ESP 250 μg i.p. alternate days over 22 days	n.d.	++	Reduced number of splenic Th1 and Th17 cells	[78]
	*F. hepatica* ESP Five or Six daily doses of 50 μg/dose i.p.	+	++	Production of innate type 2 cytokines IL-5 and IL-33.	[79]
	*Taenia Crassiceps* ESP 250 μg i.p. alternate days × 7 times	n.d.	+++	Induction of Th2. Suppression of Tumour Necrosis Factor (TNF) and IL-17. Redirected cell migration from the central nervous system to peritoneal cavity	[80]
Spinal cord homogenate in DA rats	*Trichinella spiralis* larvae ESP Multiple doses of soluble products i.p.	++	n.d.	Strong Th2-type response and increased proportion of CD4+CD25-Foxp3+ regulatory cells	[81]

^a^ SEA, Schistosome egg antigen; SP, secretory products; ESP, excretory/secretory products; i.p., intra-peritoneal injection. ^b^ The effect of helminth treatment on EAE severity, depending on the time at which helminth was administered is indicated by the following symbols: +, mild; ++, moderate; n.d., not done.

## Abbreviations

MS	Multiple sclerosis
GWAS	Genome wide association studies
ILC2	Type 2 Innate lymphoid cells
IL	Interleukin
Th	T helper
DC	Dendritic cell
Treg	T-regulatory cell
TGF-β	Transforming growth factor-beta
EAE	Experimental autoimmune encephalomyelitis
CNS	Central nervous system
MOG	Myelin oligodendrocyte glycoprotein
PLP	Proteolipid protein
MBP	Myelin basic protein
po	Oral administration
ip	Intraperitoneal injection
Sc	Subcutaneous injection
IFN	Interferon
DA	Dark Agouti
TSO	*Trichuris suis* ova
RRMS	Relapsing remitting multiple sclerosis
SPMS	Secondary progressive multiple sclerosis
MRI	Magnetic resonance imaging
NCT	National Clinical Trial
HINT	Helminth-induced immunomodulation therapy
TRIMS-A	Trichuris suis ova therapy for multiple sclerosis—a safety study
TRIOMS	Trichuris suis ova in recurrent remittent multiple sclerosis and clinically isolated syndrome
WIRMS	Worms for immune regulation of multiple sclerosis
GMP	Good manufacturing practices
SEA	Schistosoma egg antigen
ESP	Excretory secretory products
TNF	Tumour necrosis factor
NEJ	Newly excysted juveniles
FhCL	*Fasciola hepatica* cathepsin L
FhPrx	*Fasciola hepatica* peroxiredoxin
FhHDM-1	*Fasciola hepatica* helminth defense molecule
